# Skeletal-Related Events After Surgery With or Without Radiotherapy for Bone Metastases to Weight-Bearing Bones

**DOI:** 10.7759/cureus.32778

**Published:** 2022-12-21

**Authors:** Claire M Lanier, Adam G Johnson, Niema B Razavian, Joshua C Farris, Ryan T Hughes

**Affiliations:** 1 Radiation Oncology, Wake Forest School of Medicine, Winston-Salem, NC, USA; 2 Radiation Oncology, Middlesex Hospital, Middletown, CT, USA

**Keywords:** seeding, surgical fixation, post-operative radiation therapy, palliative radiation therapy, bone-metastases

## Abstract

Introduction

In patients with metastatic disease involving weight-bearing bones, postoperative radiotherapy (PORT) is commonly administered following surgical stabilization of an impending or confirmed pathologic fracture to reduce the risk of a seeded local recurrence. The goal was to re-evaluate the beneficial effect of PORT in a modern cohort of patients and determine any potential clinical predictors of skeletal-related events (SREs) which were defined as a pathologic fracture or the necessity for radiation or surgery to the affected bone.

Methods

Consecutive patients undergoing surgical stabilization of metastatic disease to weight-bearing bones of the extremities between 2012 and 2019 were reviewed. Patient, disease, and treatment factors were abstracted. The cumulative incidence of SREs was determined using competing risks methodology; overall survival (OS) was estimated using the Kaplan-Meier method.

Results

A total of 82 patients were identified, 74% of whom had undergone intramedullary nail fixation and 26% internal fixation or replacement. The femur was the most commonly involved bone (94%). A majority (78%) had an Eastern Cooperative Oncology Group (ECOG) performance status of 1-2. Bone-strengthening agents were given to 38% and PORT to 54%. The median PORT dose was 30 Gy in 10 fractions and the median percent coverage of surgical hardware was 100% (range, 25-100). SREs occurred in 10 of 82 patients. There were no differences between no RT and RT groups for the two-year cumulative incidence of SREs (8.2% vs 11.5%, p=0.59) or two-year cumulative incidence of local failure (10.8% vs 4.6%, p=0.53). The only identified predictors of SREs were the use of bone-strengthening agents (hazard ratio [HR] 0.22, 95% confidence interval [CI] 0.05-1.06, p=0.06) and malnutrition (HR 3.69, 95% CI 0.91-14.93, p=0.07). For patients treated with PORT, a biologically effective dose or percent coverage of surgical hardware was not associated with SREs.

Conclusion

In this series, the addition of PORT following surgery for metastatic disease involving weight-bearing bones does not significantly affect the rate of SREs. The use of bone-strengthening agents appears protective, and malnourished patients appear particularly at high risk for future SRE.

## Introduction

Up to 80% of solid tumor patients with advanced disease suffer from bone metastases [1]. At least half of the patients who die from cancer are thought to have bone metastases at the time of death [2]. Bone metastases are not created equally and have varying clinical presentations, implications, and treatment options. Uncomplicated, painful bone metastases without impending or existing pathologic fracture or spinal cord or cauda equina compression may be treated with radiotherapy (RT) using a variety of dose fractionation regimens [3,4]. This is particularly utilized for patients with advanced malignancy with a more limited prognosis. In the oligometastatic setting, limited bone metastases may be definitively treated using stereotactic techniques with the goal of improving progression-free survival [5,6]. Patients with metastases in weight-bearing bones such as those in the upper and lower extremities may be at risk for pathologic fracture [7]. In certain cases (for example, in patients with metastases in the lower limb or intertrochanteric region of the femur, those with >2/3 of the bone diameter involved, lytic lesions, and with pain with the use), prophylactic fixation with or without resection of the bone is recommended due to the clinical diagnosis of impending pathologic fracture [8]. 

Postoperative radiotherapy (PORT) has historically been routinely recommended after surgical fixation after retrospective case series between 1979 and 1993 observed higher rates of normal extremity use and lower rates of subsequent surgical procedures and complications in patients treated with PORT [9,10]. The hypothesized benefit was that RT to the operative site would provide a reduction in rates of local progression, hardware displacement, and re-irradiation. A more modern institutional series of patients treated with surgery followed by PORT with or without bone-strengthening agents have seemed to confirm these findings [11-13]. Thus, after surgical fixation, most guidelines recommend consideration of PORT to the involved bone in order to eradicate microscopic disease and reduce the risk of local recurrence and/or hardware failure [14-17]. However, some patients are not referred for RT consultation, usually at the discretion of the surgeon for various reasons, including the perception of limited risk of local failure, poor prognosis, distance from radiation facility/lack of transportation, or patient preference. In this study, we reviewed the records of patients treated with a surgical fixation for impending or present pathologic fracture from bone metastases in order to assess factors associated with clinical outcomes such as fracture, skeletal-related events (SREs), local control, and survival.

## Materials and methods

In this Atrium Wake Forest Baptist Health Institutional Review Board-approved investigation (approval number IRB00059805), patients with any history of malignancy treated with orthopedic surgery for prophylaxis, stabilization, or replacement of a weight-bearing extremity bone or joint were identified using a query of the electronic medical record system. If part of a patient's treatment was completed at an outside hospital (OSH), that treatment data were often unable to be reviewed. Exclusion criteria included non-malignancy indication for surgery (i.e. osteoarthritis, osteoporosis, traumatic fracture), surgery to a region of previous RT, no available medical records, biopsy only, primary malignancy of the soft tissues or bone, and age <18 years. Lymphoma was not specifically excluded but none were included in our contiguous review of bone metastasis patients. Those treated with RT versus those not treated with RT were grouped and the outcomes were compared between these strata.

SRE was defined as a pathologic fracture or necessity for radiation or surgery to the affected bone [18]. Subsequent surgery was defined as any surgical management of the affected bone or hardware associated with the initial surgery for any reason. Subsequent RT was defined as any RT that occurred greater than 180 days from the date of surgery for any reason. Local failure was defined as any radiographic evidence of disease progression in the operated bone. Overall survival (OS) was defined as the duration of time from the date of surgery to death or last follow-up. Percent coverage of surgical hardware was defined as the length of hardware in-field divided by the total length of hardware. 

Data were described using count (frequency) and median (interquartile range [IQR] or range) as appropriate and compared between groups using the chi-square test and the Wilcoxon rank sum test. OS was estimated using the Kaplan-Meier method. Cumulative incidence rates of SREs and local failure were estimated using competing risk methodology (death without event as a competing risk) [19]. Statistical analyses were performed using R version 3.6 (R 83 Foundation for Statistical Computing, Vienna, Austria) [20].

## Results

In total, 123 patients were identified. A total of 41 patients were excluded for the following reasons: benign indication for surgery (n=23), prior RT (n=13), no records (n=2), biopsy only (n=1), non-bone metastasis indication (n=1), and pediatric (n=1). This resulted in a cohort of 82 patients. Patient and treatment factors are summarized in Table 1.

**Table 1:** Patient Characteristics and Treatment Factors IMN, intramedullary nail; ECOG, Eastern Cooperative Oncology Group; DM, diabetes mellitus; HTN, hypertension; PORT, postoperative radiation therapy; OSH, outside hospital, IQR, interquartile range; BMI, body mass index. N represents the total number of patients.

Patient characteristics		No of patients (N=82)
Gender (n (%))		
	F	38 (46.3)
	M	44 (53.7)
Surgery type (n (%))		
	Fixation/Replacement	21 (25.6)
	IMN	61 (74.4)
Primary (n (%))		
	Breast	21 (25.6)
	Lung	20 (24.4)
	Prostate	8 (9.8)
	Other	33 (40.2)
ECOG (n (%))		
	1	37 (45.7)
	2	26 (32.1)
	3	17 (21.0)
	4	1 (1.2)
DM (n (%))		
	No	52 (63.4)
	Yes	30 (3.6)
HTN (n (%))		
	No	28 (34.1)
	Yes	54 (65.9)
Malnutrition (n (%))		
	No	67 (81.7)
	Yes	15 (18.3)
Smoking (n (%))		
	No	56 (68.3)
	Yes	26 (31.7)
Surgical site (n (%))		
	Femur	77 (93.9)
	Humerus	4 (4.9)
	Tibia	1 (1.2)
Prophylactic surgery (n (%))		
	No	22 (26.8)
	Yes	60 (73.2)
Bone agent (n (%))		
	No	51 (62.2)
	Yes	31 (37.8)
Type of bone agent (n (%))		
	None	51 (62.2)
	Denosumab	24 (29.3)
	Zolendronic acid	6 (7.3)
	Denosumab and zoledronic acid	1 (1.2)
PORT (n (%))		
	No	38 (46.3)
	Yes	44 (53.7)
Hardware covered (n (%))		
	Yes	23 (51.1)
	No	6 (13.3)
	OSH	16 (35.6)
Age (median [IQR])	66.00 [57.25, 73.00]	
BMI (median [IQR])	27.46 [23.71, 29.55]	

A majority of patients had a breast primary followed by lung or prostate. There was also a heterogeneous group of other less common cancers comprising 40% of all cases. Other cancers included neuroendocrine tumors, carcinoid tumors, and cutaneous squamous cell carcinomas (SCCs). The site of origin/histology was unknown in four cases. There were no lymphoma patients reviewed in this study although not specifically excluded. The majority of patients treated with RT had undergone an intramedullary nail (IMN) procedure (74%) rather than a fixation or replacement of the affected bone (26%). For patients not treated with PORT, 66% had undergone IMN and 34% were treated with fixation/replacement. Bone-strengthening agents such as zoledronic acid and/or denosumab were utilized in 52% of those in the PORT group compared to 21% in the no PORT group (p=0.01). There were no significant differences between no RT and RT groups for age, gender, comorbidity, preoperative opioid requirement, involved bone, primary malignancy, or preoperative Eastern Cooperative Oncology Group (ECOG).

Median follow-up for all patients was 8.1 months (range: 0.03-81.4). In total, 10 SREs were observed, 4 of 38 (11%) in the no PORT group and 6 of 44 (14%) in the PORT group (p=0.93). Subsequent surgery to the site of initial fixation was required in a total of 9 (11%) patients, 4 of 38 in the no PORT group, and 5 of 44 in the PORT group. Re-irradiation for recurrent pain was delivered in only 1 of 44 patients. Local failure in the involved bone was observed in four and three patients in the no PORT and PORT groups, respectively. There were no differences in SREs or narcotic use (morphine equivalent daily dose before surgery versus at last follow-up) between groups as noted in Table 2.

**Table 2:** Outcomes PORT, postoperative radiation therapy; MEDD, morphine equivalent daily dose; IQR, interquartile range. N represents the total number of patients.

Patient characteristics		Overall (N=82)	No PORT (N=38)	PORT (N=44)	p-Value
Subsequent surgery (n (%))					1
	No	73 (89.0)	34 (89.5)	39 (88.6)	
	Yes	9 (11.0)	4 (10.5)	5 (11.4)	
Subsequent radiation therapy (n (%))					1
	No	81 (98.8)	38 (100.0)	43 (97.7)	
	Yes	1 (1.2)	0 (0.0)	1 (2.3)	
Skeletal-related event (n (%))					0.93
	No	72 (87.8)	34 (89.5)	38 (86.4)	
	Yes	10 (12.2)	4 (10.5)	6 (13.6)	
Local failure (n (%))					0.84
	No	75 (91.5)	34 (89.5)	41 (93.2)	
	Yes	7 (8.5)	4 (10.5)	3 (6.8)	
Preoperative MEDD (median [IQR])		45.00 [0.00, 102.50]	47.00 [0.00, 154.50]	30.00 [0.00, 90.00]	0.21
MEDD at last follow-up (median [IQR])		60.00 [17.50, 120.00]	60.00 [30.00, 91.50]	52.50 [9.00, 138.75]	0.79
Change in MEDD (median [IQR])		0.00 [-7.00, 60.00]	10.00 [-35.25, 51.00]	0.00 [-2.00, 63.00]	0.64

**Figure 1:**
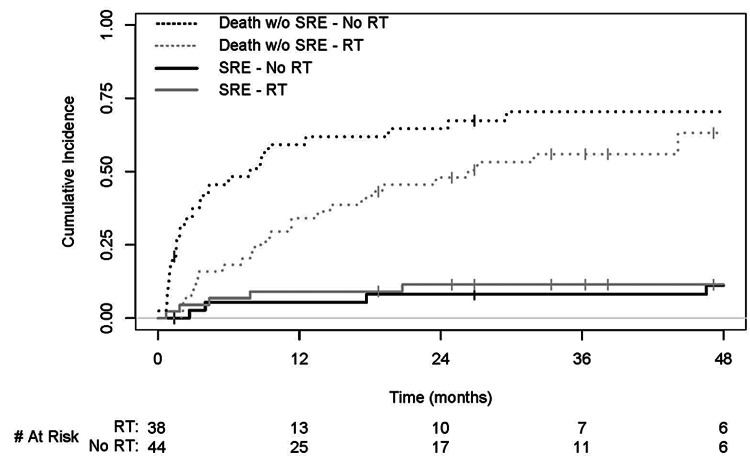
Cumulative Incidence of Skeletal-Related Events With Death as a Competing Risk in Patients Treated With and Without Postoperative Radiotherapy RT, radiotherapy.

The overall one- and two-year cumulative incidence rates of SREs were 7.4% (95% confidence interval [CI]: 1.7-13.1) and 9.9% (95% CI: 3.4-16.4), respectively. The corresponding cumulative incidence rates of the competing risk (death without SREs) were 45.6% (95% CI: 34.8-56.5) and 55.6% (95% CI: 44.8-66.5), respectively. Comparing the no RT and RT groups, the one-year cumulative incidence of SREs was 5.4% (95% CI: 3.7-12.8) versus 9.1% (95% CI: 6.0-17.6) and the two-year cumulative incidence was 8.2% (95% CI: 4.5-17.0) versus 11.5% (95% CI: 2.0-21.0), respectively. There was no difference in the cumulative incidence of SREs between no RT and RT groups (Gray’s p=0.59). Cumulative incidence plots of SREs with death as a competing risk by treatment group are shown in Figure 1.

Various patient, disease, and treatment-related factors were tested for association with SREs and no tested factors were associated with SREs on bivariate analysis as shown in Table 3. There was a trend toward bone agents being protective against SREs and malnutrition being a risk for SREs but neither finding was significant. The most common radiation regimen was 30 Gy in 10 fractions at 3 Gy per fraction.

**Table 3:** Bivariate Analysis of Factors Related to Skeletal-Related Events SRE, skeletal-related events; IQR, interquartile range; BMI, body mass index; IMN, intermedullary nail; ECOG, Eastern Cooperative Oncology Group; DM, diabetes mellitus; HTN, hypertension; OSH, outside hospital. N represents the total number of patients.

Patient characteristics		Patients without SREs (N=72)	Patients with SREs (N=10)	p-Value
Age (median [IQR])		66.00 [58.00, 73.00]	65.50 [56.25, 71.00]	0.799
BMI (median [IQR])		27.79 [23.90, 29.88]	26.65 [21.95, 28.77]	0.47
Radiation dose (median [IQR])		30.00 [30.00, 30.00]	30.00 [30.00, 30.00]	0.213
Number of fractions (median [IQR])		10.00 [10.00, 10.00]	10.00 [10.00, 10.00]	0.213
Gender (n (%))				0.443
	F	35 (48.6)	3 (30.0)	
	M	37 (51.4)	7 (70.0)	
Surgery type (n (%))				1
	Fixation/Replacement	18 (25.0)	3 (30.0)	
	IMN	54 (75.0)	7 (70.0)	
Primary (n (%))				0.207
	Breast	20 (27.8)	1 (10.0)	
	Lung	19 (26.4)	1 (10.0)	
	Other	26 (36.1)	7 (70.0)	
	Prostate	7 (9.7)	1 (10.0)	
ECOG (n (%))				0.976
	1	32 (45.1)	5 (50.0)	
	2	23 (32.4)	3 (30.0)	
	3	15 (21.1)	2 (20.0)	
	4	1 (1.4)	0 (0.0)	
DM (n (%))				0.912
	No	45 (62.5)	7 (70.0)	
	Yes	27 (37.5)	3 (30.0)	
HTN (n (%))				1
	No	25 (34.7)	3 (30.0)	
	Yes	47 (65.3)	7 (70.0)	
Malnutrition (n (%))				0.558
	No	60 (83.3)	7 (70.0)	
	Yes	12 (16.7)	3 (30.0)	
Smoking (n (%))				1
	No	49 (68.1)	7 (70.0)	
	Yes	23 (31.9)	3 (30.0)	
Surgical site (n (%))				0.68
	Femur	68 (94.4)	9 (90.0)	
	Humerus	3 (4.2)	1 (10.0)	
	Tibia	1 (1.4)	0 (0.0)	
Prophylactic surgery (n (%))				1
	No	19 (26.4)	3 (30.0)	
	Yes	53 (73.6)	7 (70.0)	
Bone agent (n (%))				0.373
	No	43 (59.7)	8 (80.0)	
	Yes	29 (40.3)	2 (20.0)	
Hardware covered (n (%))				0.25
	No	4 (10.3)	2 (33.3)	
	Yes	20 (51.3)	3 (50.0)	
	OSH	15 (38.5)	1 (16.7)	

Two-year cumulative incidence of local failure was 10.8% (95% CI: 0.8-20.8) in the no RT group compared with 4.6% (95% CI: 3.1-10.7) in the RT group (Gray’s p=0.53). Of the three patients treated with PORT who experienced a local failure, the percent coverage of the hardware was 33%, 75%, and 100%.

**Figure 2:**
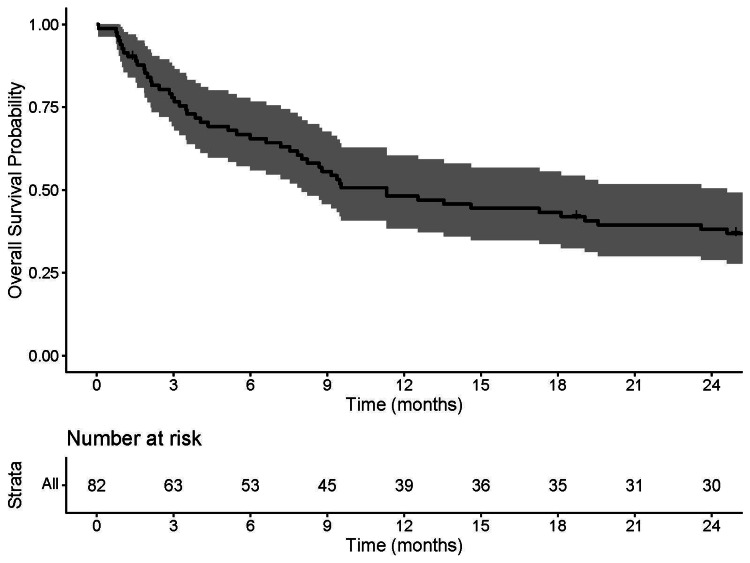
Overall Survival of the Entire Cohort

Median OS for the entire cohort was 11.3 months (95% CI: 8.0-24.6). Death within three months of surgery occurred in 14 of 38 (36.8%) patients not treated with PORT compared with 4 of 44 (9.1%) patients treated with RT (p<0.01). The Kaplan-Meier estimate of three-month OS was 77.9% (95% CI: 69.4-87.4); OS at one year was 48.2% (95% CI: 38.5-60.4) as shown in Figure 2.

## Discussion

The routine practice of PORT in the setting of bone metastases undergoing prophylactic or therapeutic fixation is based on limited retrospective data suggesting a benefit with regard to limb use and hardware complications that may necessitate subsequent surgical intervention. However, this has yet to be confirmed in large, prospective studies; the clinical outcomes impacted and the magnitude of such effect are unclear. In this study, the one-year freedom from SRE was not statistically different for those who did versus did not receive PORT, respectively. These findings suggest that PORT may not be beneficial for all patients after surgical fixation. Consensus guidelines are based on historical retrospective studies which identified a clinical benefit in patients treated with PORT [9]. While not directly investigating the occurrence of SRE, they did look into achieving and maintaining a normal functional status and the need for additional orthopedic procedures which were all improved with PORT [9]. An improvement in OS was also observed with PORT [9]. It is likely that selection bias resulted in patients with more favorable prognosis and/or functional status being referred for PORT. Our results demonstrated a 37.8% rate of death within three months of surgery in patients not treated with RT compared to 8.9% in patients treated with RT, suggesting that this may be affecting our sample as well. As PORT is considered the standard of care, much of the research has related to the ideal dose and fractionation, which is also unclear in the literature. Two recent studies indicate different results, specifically that a single fraction is equivalent to multiple fractions [21] and, alternatively, that PORT using 30 Gy in 10 fractions is preferred [22].

It is very possible that advances in systemic therapy affect the potential benefit of PORT with regard to the prevention of SRE. Bone-modifying agents are considered the standard of care for patients with metastatic breast cancer as well as those with bone metastases from any solid tumor [19,20]. In fact, a randomized controlled clinical trial comparing placebo with zoledronic acid demonstrated a reduced incidence of SRE in men with metastatic prostate cancer [23,24]. In our study, 38% of patients were treated with bone-strengthening agents, and those treated with PORT were significantly more likely to have also been receiving bone-strengthening agents than those not treated with PORT. While these agents have been shown to reduce the rates of SRE in patients with multiple tumor types, it is unclear whether their effects provide a local benefit after a surgical fixation with regard to hardware stabilization, subsequent surgery, or pain. It is unclear if these agents would prevent a local failure in the involved bone and PORT remains a clear consideration when there is a concern for a high risk of local recurrence, particularly after a multidisciplinary discussion.

With advances in cancer care and improved OS in patients with metastatic cancer, bone metastases and their ideal treatment are more relevant than ever. A prospective, randomized trial evaluating patient outcomes in those treated surgically plus or minus radiation, with single versus multifraction radiation regimens, is needed to identify a definitive recommendation for this growing patient population. This is currently under investigation in the Dutch PORT trial (NCT02705183).

There were several limitations to this study as well. Given its retrospective nature, limited sample size and various biases that are difficult to assess and adjust for likely impact these findings. Outcome data, particularly related to pain outcomes, are difficult to collect and control for using a retrospective observational design.

## Conclusions

In this study of patients with metastatic disease involving weight-bearing bones treated with a surgical fixation for impending or present pathologic fracture, we found no difference in the rates of SREs in those treated with versus without PORT. Of note, the use of bone-strengthening agents continues to appear protective and malnourished patients are at higher risk of future SREs and may require special consideration. Further studies need to be performed, ideally a randomized controlled trial investigating surgery plus or minus PORT in one or more fractions. As novel cancer treatments continue to extend the OS in patients with metastatic cancer, the optimal treatment of pathologic fractures of the long bones needs further exploration.
